# Elite Athletes’ Experiences During Injury Rehabilitation from a Biopsychosocial Perspective: An Exploratory Qualitative Study

**DOI:** 10.3390/bs16081361

**Published:** 2026-08-09

**Authors:** Moonjung Bae

**Affiliations:** Biomedical Research Institute, H+ Yangji Hospital, Seoul 08779, Republic of Korea; m4222@newyjh.com; Tel.: +82-70-4665-9174

**Keywords:** sports injury, rehabilitation, biopsychosocial experience, qualitative analysis

## Abstract

Elite athletes experience diverse biological, psychological, and social challenges during injury rehabilitation, yet few qualitative studies have explored these experiences and their interactions from a biopsychosocial perspective. This exploratory qualitative study investigated the biopsychosocial experiences of elite athletes during injury rehabilitation and the interactions among these experiences through semi-structured interviews with five participants, using inductive content analysis. Athletes reported biological experiences including musculoskeletal symptoms, limitations in daily activities, adverse drug reactions, psychosomatic symptoms, and physical development; psychological experiences including negative emotions (anger, anxiety, and depression) and positive experiences (restoration and psychological resilience); and social experiences including informational, instrumental, and emotional support, as well as negative social influences such as blame and pressure to return to play. Participants perceived that these biological, psychological, and social experiences were closely interconnected throughout rehabilitation. Biological experiences, such as pain and physical impairment, were perceived to contribute to negative emotions, including anxiety, whereas negative emotions were associated with stress-related psychosomatic symptoms, such as gastrointestinal symptoms and headaches. Emotional support was associated with positive emotional experiences, whereas negative social influences, including blame and pressure to return to play, were associated with negative emotional responses. These findings contribute to an understanding of the multidimensional experiences of injured elite athletes and may inform development of integrated rehabilitation approaches that address biological, psychological, and social aspects of recovery.

## 1. Introduction

Injuries are an inevitable challenge faced by elite athletes, impacting not only their athletic performance but also their overall quality of life. Elite athletes commonly experience acute injuries resulting from unexpected contact or movements during competition (Engebretsen et al., 2013; Soligard et al., 2017), as well as overuse injuries caused by high-intensity repetitive training (Franco et al., 2021). Depending on the severity of the injury, surgical intervention may be necessary, and the rehabilitation period typically extends over a prolonged duration, often ranging from 6 to 12 months.

During injury rehabilitation, athletes experience biological, psychological, and social challenges that collectively influence rehabilitation and return-to-play outcomes (Brewer & Redmond, 2017). Following a sports injury, athletes commonly experience physical impairments, including pain, muscle atrophy resulting from immobilization, and reduced joint range of motion (Akeson et al., 1987; Appell, 1990; Ji & Yeo, 2019). Delays in physical recovery during rehabilitation may increase athletes’ anxiety about recovery and return to performance, and such anxiety may, in turn, influence rehabilitation outcomes (Forsdyke et al., 2016; Wiese-Bjornstal et al., 1998).

In addition to the psychological impact of delayed physical recovery, injured athletes commonly experience negative emotional responses such as anxiety, depression, and low self-esteem throughout the rehabilitation process (Leddy et al., 1994). Although positive emotions, including hope and optimism, may emerge as recovery progresses, fear of reinjury and concerns about return to sport often persist (Clement et al., 2015). Systematic reviews have demonstrated that psychological factors such as anxiety, fear, confidence, and motivation significantly influence rehabilitation outcomes (Ardern et al., 2013; Forsdyke et al., 2016).

Socially, athletes receive emotional, informational, and instrumental support throughout the rehabilitation process. Emotional and instrumental support are provided primarily by friends and family members, whereas informational support related to injury rehabilitation is typically obtained from physical therapists, medical professionals, and fellow athletes with prior injury experience (Johnston & Carroll, 1998). Emotional support has been shown to positively influence the reduction in negative emotions in athletes recovering from concussion (Wayment & Huffman, 2020). Conversely, the loss of one’s role within the team or excessive expectations regarding return to play may adversely affect rehabilitation outcomes (Liu et al., 2025; Wiese-Bjornstal et al., 1998).

Collectively, previous studies suggest that biological, psychological, and social factors are all important determinants of rehabilitation outcomes and should be considered together within a biopsychosocial perspective (Bae, 2024; Brewer & Redmond, 2017). This interconnected nature of injury rehabilitation is reflected in the integrated model of psychological responses to sport injury, which proposes that athletes cognitively appraise biological aspects of their injury, such as injury severity and functional limitations, and that these appraisals subsequently influence their emotional and behavioral responses to rehabilitation (Wiese-Bjornstal et al., 1998). Furthermore, this dynamic process is shaped by both personal factors, such as injury history and athletic identity, and social factors, including support and influences from family members, teammates, coaches, and healthcare professionals. Accordingly, biological, psychological, and social factors continuously interact throughout the rehabilitation process rather than functioning as independent domains.

Therefore, injury rehabilitation should be understood as a dynamic biopsychosocial process rather than as a series of isolated biological, psychological, and social experiences. However, most previous studies have examined biological, psychological, and social domains separately, and relatively few qualitative studies have explored how elite athletes perceive the dynamic interactions among these experiences throughout injury rehabilitation. Accordingly, this exploratory qualitative study sought to explore the biological, psychological, and social experiences of elite athletes during injury rehabilitation through in-depth interviews. In addition, the study aimed to explore how participants perceived the interactions among these experiences throughout the rehabilitation process. The findings may provide a deeper understanding of the biopsychosocial rehabilitation process and inform the development of more comprehensive rehabilitation support systems and return-to-play strategies. The research questions for this study are as follows.

1. What are the biological, psychological, and social experiences of elite athletes during injury rehabilitation?
2. How are elite athletes’ biological, psychological, and social experiences interconnected during injury rehabilitation?

## 2. Materials and Methods

### 2.1. Study Design

This study employed an exploratory qualitative research design to gain an in-depth understanding of the biopsychosocial experiences of elite athletes during the injury rehabilitation process. To this end, in-depth interviews were conducted with elite athletes who had experienced injury rehabilitation. The interview data were analysed via inductive content analysis, which allowed the identification of key biological, psychological, and social themes related to their rehabilitation experiences. This study was approved by the Institutional Review Board of H+ Yangji Hospital (IRB No. HYJ 2024-12-020). All participants provided written informed consent prior to participation in the study, in accordance with the Declaration of Helsinki.

### 2.2. Participants

This study conducted in-depth interviews with five elite athletes enrolled in a sports university in Seoul who had experienced injury rehabilitation. In this study, elite athletes were defined as athletes registered with sport organizations recognized by the Korea Sports & Olympic Committee and who had experience participating in national-level competitions. The specific inclusion and exclusion criteria are presented in Table 1. Athletes were included if they had been removed from training or competition for more than three months due to injury and had undergone rehabilitation for at least three months. Athletes who declined to participate in the study were excluded.

This study adopted an exploratory qualitative research design aimed at exploring elite athletes’ biological, psychological, and social experiences during injury rehabilitation rather than generalizing the findings to a broader population. In qualitative research, participant recruitment is guided by information richness and thematic sufficiency rather than predetermined numerical criteria (Braun & Clarke, 2013). Accordingly, participants were purposively recruited to provide rich accounts of injury rehabilitation experiences. Recruitment continued until additional interviews yielded no substantially new insights relevant to the study aims. Given the exploratory nature of this study and the relatively small sample, the findings should be interpreted as preliminary and are not intended for statistical or population-level generalization.

This study employed purposive sampling to recruit participants. The author (MJ) visited a sports university in Seoul to identify eligible participants based on predefined inclusion and exclusion criteria and provided detailed information on the study purpose and procedures. Participants who met the selection criteria were invited to take part in in-depth interviews. Subsequently, snowball sampling was used, whereby participants recommended other eligible athletes. No eligible participants who were approached declined to participate in the study. Through this process, a total of five participants were recruited. The interviewer had no prior relationship with the participants. In-depth interviews were conducted only with individuals who provided written informed consent. To ensure anonymity, participants were assigned identification codes (I001, I002, I003, I004, and I005). The demographic characteristics and injury-related information of the participants are presented in Table 2. All participants met the predefined criteria for elite athletes described in this study. Although participants were recruited through a sports university, all participants were graduate students and were living independently rather than in shared athletic accommodation at the time of the interviews.

### 2.3. Data Collection

This study employed in-depth interviews to explore the biopsychosocial experiences of elite athletes during the injury rehabilitation process. Prior to the interviews, the purpose of the study was explained in detail, and interviews were conducted only with participants who provided informed consent. All interviews took place in a private, confidential setting to ensure the participants’ privacy.

In-depth interviews were conducted using a semi-structured interview format. Each interview began with the open-ended questionnaire items, followed by additional probing questions based on the participant’s responses. This semi-structured approach allowed for both consistency and flexibility in exploring individual experiences. The semi-structured interviews were guided by the following open-ended questions:

*Please describe your experiences during the injury rehabilitation process. You may include any experiences related to your body, emotions, relationships with others, or any other experiences that you consider meaningful.*

The in-depth interviews were conducted directly by the author (MJ). The author is a family medicine physician with experience as a national team doctor and has treated numerous injuries in elite athletes for several years. Through this clinical experience, the author developed a deep interest in the challenges athletes face during injury rehabilitation and has conducted related research (Bae, 2024; Yoon et al., 2018). Additionally, as a Ph.D. holder in sport psychology, the author has received formal training in qualitative research and has participated in multiple qualitative studies.

Although the researcher’s professional background as a family medicine physician, national team physician, and PhD-trained sport psychologist facilitated an in-depth understanding of sports injury rehabilitation, it also introduced the potential for researcher bias during data collection and interpretation. To minimize this possibility, the researcher maintained a non-judgmental stance throughout the interviews, encouraged participants to express their experiences freely, and explained that their responses would remain confidential and would not influence their academic evaluation or athletic status.

In addition, emerging codes and interpretations were regularly discussed with an independent peer debriefer (HB) to enhance the credibility and trustworthiness of the findings. Although HB was not listed as an author of this study, HB contributed to the data analysis process by serving as an independent peer debriefer for cross-checking emerging codes and interpretations. HB is a former elite athlete, holds a Ph.D. in sport psychology, and is currently a professor at a college of physical education, providing relevant expertise to support the independent review of the qualitative analysis.

Each in-depth interview was conducted once and lasted approximately 60 min. When additional information was needed, it was obtained through follow-up communication via face-to-face meetings, phone calls, or messaging. But no follow-up interviews were conducted after the initial interview. All interviews were audio-recorded using a digital voice recorder, and the recordings were transcribed verbatim into Microsoft Word documents. The transcribed content was shared with the participants to confirm the accuracy of the data.

### 2.4. Data Analysis

The audio recordings of the in-depth interviews were transcribed into Microsoft Word files. Interviews were conducted and recorded in Korean, the native language of the participants. The interview data and quotations included in the manuscript were translated into English by researcher MJ, who is a native Korean speaker with expertise in sport psychology and sports medicine and is fluent in English. Repeated reviews were conducted during translation to preserve contextual and semantic accuracy. In addition, the translated interview data and quotations were independently reviewed by a colleague fluent in both Korean and English to verify the accuracy and consistency of the translations.

The transcribed data were repeatedly read and segmented into meaningful units. Each unit was coded using appropriate psychological terms relevant to the research topic. Subsequently, inductive categorization was performed by grouping the coded units into thematically similar categories. Through this process, key themes related to the biopsychosocial experiences central to the study were identified.

Although the study was conceptually informed by the biopsychosocial framework, the coding process was conducted inductively. Initial codes and themes were generated directly from participants’ narratives without using predefined categories (Table 3). After the inductive analysis had identified themes, these themes were subsequently organized and interpreted within the biopsychosocial framework for conceptual presentation and discussion. Thus, the biological, psychological, and social perspectives served as an organizing framework for the final findings rather than as a predetermined coding framework. The coding and inductive categorization were carried out by two sport psychology researchers (MJ and HB). The results were cross-validated by both researchers, and any discrepancies were resolved through discussion until consensus was reached. The process of inductive categorization of the data is presented in Table 3.

## 3. Results

### 3.1. Biopsychosocial Experience During Injury Rehabilitation

The analysis identified eleven themes describing the injury rehabilitation experiences of elite athletes. These themes were subsequently organized into biological, psychological, and social perspectives within the biopsychosocial framework (Table 4).

#### 3.1.1. Theme 1: Musculoskeletal Symptoms

Athletes reported experiencing a variety of musculoskeletal symptoms during injury rehabilitation. The most commonly reported symptom was pain, which was associated with the injury site, surgical area, or rehabilitation exercises. Participants described persistent discomfort that interfered with movement and daily activities. One athlete described the pain and functional limitations experienced during rehabilitation:

*“During the rehab period, I kept feeling pain where the metal pin from the surgery was inserted, and it was quite uncomfortable.”*

(I001)

In addition to pain, athletes frequently reported muscle atrophy resulting from prolonged inactivity and immobilization. Participants noted that muscle mass decreased rapidly following injury or surgery and that regaining lost strength required a considerably longer period of rehabilitation. As one participant explained:

*“For about six weeks after the surgery, I couldn’t walk properly, and I lost a lot of muscle during that time. What was frustrating was that regaining that muscle took way longer than those six weeks.”*

(I004)

Some athletes further reported asymmetry between the injured and uninjured sides of the body. Participants perceived that compensatory movement patterns resulting from the injury contributed to postural imbalances and altered body alignment. As one athlete explained:

*“I thought I was walking normally, but because my knee was misaligned, my pelvis became misaligned, and then my shoulders became misaligned as well. As a result, I felt that my whole body had become asymmetrical and unbalanced.”*

(I001)

#### 3.1.2. Theme 2: Limitations in Daily Activities

During the rehabilitation period, athletes experienced various limitations in their daily lives. Pain, muscle weakness, and reduced physical function associated with the injury interfered with routine activities such as walking, climbing stairs, carrying objects, and maintaining certain postures. Participants described these limitations as frustrating because tasks that had previously been performed without difficulty became physically demanding. One athlete explained the challenges associated with lower back pain:

*“When I injured my back, even taking a shower or washing my face felt really difficult. Since I play a team sport and live in shared accommodations, it was tough because I ended up having to rely on help from my teammates, which wasn’t easy for me.” *

(I005)

The other participant stated:

*“Compared to before the injury, my knee gets tired much more easily, and the soreness is worse. Especially when I sit on the floor, it’s really hard to stay seated for a long time.”*

(I003)

#### 3.1.3. Theme 3: Psychosomatic Symptoms

Some athletes reported experiencing physical symptoms that they perceived to be associated with psychological stress during injury rehabilitation. Commonly reported symptoms included headaches, gastrointestinal discomfort, and stress-induced binge eating. Several participants described experiencing persistent headaches, which they attributed to ongoing psychological stress and concerns regarding their recovery. One athlete stated:

*“Because I was constantly worrying about things, I think I started frowning and getting tense without even realizing it, so I ended up having frequent headaches all the time.”*

(I001)

Athletes also reported gastrointestinal symptoms, including indigestion, heartburn, gastritis, and abdominal discomfort. Participants perceived that these symptoms worsened during periods of heightened stress. As one participant explained:

*“I think the rehab period was when I felt the most stressed. The stress really affected my stomach, I couldn’t digest food properly, and the headaches were incredibly intense.”*

(I005)

One athlete further described stress-induced binge eating as a coping behavior during rehabilitation. The participant reported repeatedly consuming spicy and greasy foods when experiencing stress, which was followed by recurrent gastrointestinal discomfort.

*“When I was stressed, I’d eat spicy or greasy food, and the next day my stomach would hurt and feel really raw… But then when I got stressed again, I’d go back to eating those same kinds of foods. I think I just kept repeating that cycle.”*

(I001)

#### 3.1.4. Theme 4: Adverse Drug Reactions

Some athletes reported experiencing adverse drug reactions during the rehabilitation period, particularly in response to medications such as analgesics, nonsteroidal anti-inflammatory drugs (NSAIDs), and glucocorticoids. The most commonly reported side effects were gastrointestinal symptoms, including heartburn and indigestion. These symptoms occurred after medication use and were identified as adverse drug reactions based on their temporal relationship to drug administration. One participant described persistent gastrointestinal discomfort associated with prolonged use of prescribed pain medication:

*“Every time I took my medication, I felt a burning sensation in my stomach… but I had to take it anyway. So there were many times when I just had to put up with the discomfort and take the medicine.”*

(I001)

#### 3.1.5. Theme 5: Physical Development

Athletes reported experiencing physical development throughout the rehabilitation process. Through rehabilitation training, participants perceived improvements in muscle strength and physical function that had been diminished by injury. Some athletes reported that the rehabilitation process enabled them to identify previously weak muscle groups and address these deficiencies through targeted exercises. One athlete described how rehabilitation contributed to overall physical development:

*“Through rehab, I started paying more attention to taking care of my body. I became more aware of which muscles were weak and, based on the demands of my sport, I learned which specific muscles I needed to strengthen—and I worked on building those up”*

(I002)

The other participant explained:

*“I used to have pretty weak hamstrings, but during rehab, I focused on strengthening my hamstrings and glute muscles. Once those muscles got stronger, my speed noticeably improved. I also felt a big difference in my explosiveness—like in tackles—and even my shooting got better.”*

(I005)

#### 3.1.6. Theme 6: Negative Emotions

Most athletes experienced anxiety during the rehabilitation period. Participants commonly reported uncertainty regarding the recovery process and concerns about whether they would be able to return to their preinjury condition. Delays in recovery or persistent symptoms often intensified these feelings of uncertainty. One athlete described anxiety associated with ongoing symptoms and concerns about recovery:

*“There was always a warm sensation around the surgical site. Because of that, I kept worrying… ‘What if it’s getting inflamed again?’ or ‘What if my recovery is slower than it should be?’ Those thoughts made me feel anxious.”*

(I001)

Athletes also expressed concerns regarding their future athletic careers and the possibility that they might not regain their previous level of performance. In some cases, these concerns extended to fears of retirement. As one participant explained:

*“Since the injury just wasn’t getting better, I started thinking, ‘What if I can’t do fencing anymore?’ and ‘What if I end up having to retire unexpectedly?’ Those thoughts made me feel really uneasy.”*

(I002)

Athletes experienced profound feelings of depression during the rehabilitation period, largely due to a sense of isolation from teammates and disconnection from training and competition. Being unable to participate in team activities often led to feelings of loneliness and social exclusion. One athlete described these experiences as follows:

*“During rehab, while my teammates were heading off to train, I had to leave for rehab sessions outside. I felt left out, and I couldn’t help but wonder, ‘Why is this happening to me?’ It made me feel really down and even depressed at times.”*

(I001)

Some athletes also reported that prolonged rehabilitation and uncertainty regarding recovery contributed to feelings of helplessness and emotional exhaustion. As one participant explained:

*“Since I couldn’t do any fencing training with the team anyway, I stayed in Seoul to focus solely on rehab. But even after a few months, the pain in the injured area just wouldn’t go away, and that made me feel really depressed.”*

(I002)

The team sport athlete felt isolated and depressed while undergoing rehabilitation, as the other athletes laughed and enjoyed training together whereas they had not yet fully recovered. As one participant explained:

*“Living in shared housing with other athletes, I’d see them laughing and having fun, but I just didn’t feel any joy. Being in pain made me think more negatively, and I felt really down. That sadness ended up coming out as frustration, and I found myself taking it out on my parents.”*

(I005)

Athletes reported experiencing anger during the rehabilitation period. Many expressed frustration regarding the unexpected nature of their injuries and the disruption of their athletic goals. For some athletes, the injury itself was perceived as an unfair obstacle that interrupted their progress and development. One athlete described feeling anger toward the injury and its consequences:

*“I kept asking myself, ‘Why do these injuries keep happening to me?’ And since it ended up being serious enough to require surgery, I started thinking, ‘Maybe I’m just not meant to succeed as an athlete.’ That thought made me really angry.”*

(I001)

Several athletes also reported directing anger toward themselves, blaming themselves for failing to prevent the injury. As one participant explained:

*“When I got injured, I was angry at myself, thinking, ‘Why didn’t I prepare for something like this?’ I couldn’t stop blaming myself for not seeing it coming.”*

(I004)

#### 3.1.7. Theme 7: Positive Emotions

Although most athletes reported negative emotional experiences during rehabilitation, some perceived the rehabilitation period as an opportunity for physical and psychological recovery. Participants described rehabilitation as a temporary break from the demands of training and competition, allowing them to recover from accumulated fatigue and engage in self-reflection. One athlete explained:

*“While other teammates were undergoing intense daily training, I wasn’t training at that level during the rehabilitation period. As a result, my body felt relatively more comfortable, and I came to perceive it somewhat as a period of rest.”*

(I001)

Some athletes reported experiencing psychological resilience during the rehabilitation period, demonstrating the ability to adapt positively to adversity. Although they initially experienced frustration and anxiety following injury, the rehabilitation process provided opportunities for personal growth and self-reflection. Several athletes described adopting a positive perspective toward rehabilitation, viewing it as an opportunity to rebuild themselves physically and mentally. One participant explained:

*“I’m usually a pretty positive person, so I told myself, ‘Let’s just focus on rehab and take this time to really rebuild my body.’”*

(I003)

Athletes also reported gaining a deeper understanding of their bodies and identifying areas for improvement through rehabilitation. Rather than viewing rehabilitation solely as recovery from injury, some perceived it as an opportunity for long-term development. As one participant stated:

*“Seeing other athletes come back after injuries made me feel like, of course, I could do it too. Since I started my sport later than most, my form wasn’t perfect, but I took this as a chance to erase those bad habits and start fresh—approaching rehab with the mindset of rebuilding from the ground up.”*

(I004)

In addition, progress during rehabilitation fostered hope and confidence regarding recovery and return to sport. As symptoms improved, athletes reported increased motivation to actively engage in the rehabilitation process. One athlete noted:

*“During rehab, there was this moment when, all of a sudden, I didn’t feel any pain—and that’s when I started to see a glimmer of hope. It really motivated me to push even harder with my rehab.”*

(I002)

#### 3.1.8. Theme 8: Instrumental Support

Elite athletes without team affiliation reported financial difficulties in covering the costs of injury rehabilitation because they lacked a stable income. Consequently, they received financial support from their parents to cover expenses such as surgery, medical consultations, and rehabilitation treatments. One participant explained: *“Since I wasn’t part of a professional team at the time, my parents covered all the rehab treatment costs.”* (I005). In contrast, some athletes affiliated with a team reported receiving partial financial support for rehabilitation-related expenses from their teams. As one participant stated: *“The team provided some support by covering part of my rehab treatment costs.”* (I002). In addition, the athletes also received rehabilitation equipment from their teammates. One participant described this experience as follows: *“As for things like braces, I was able to use ones that my teammates weren’t using—they let me borrow them.”* (I001).

#### 3.1.9. Theme 9: Informational Support

For injured athletes, accessing a reputable sports medicine hospital or rehabilitation center specialized in their specific type of injury was considered essential. Participants frequently reported receiving recommendations from coaches, teammates, or senior athletes who had previously experienced injury rehabilitation. These recommendations helped reduce uncertainty when selecting medical institutions and increased confidence in the treatment and rehabilitation process. One athlete described being referred to a rehabilitation center that specialized in treating athletes from the same sport:

*“A close junior teammate introduced me to a rehab center that specializes in working with fencing athletes. The therapist there had been treating fencers for over 10 years and was even learning fencing themselves. Because he really understood the sport, he was able to teach me rehab exercises that were perfectly suited to my needs.”*

(I002)

Athletes who experienced injury for the first time often reported feelings of anxiety due to uncertainty surrounding the rehabilitation process. Informational support from senior athletes, teammates, and coaches who had previously experienced similar injuries helped reduce this uncertainty. Participants reported receiving practical advice regarding rehabilitation exercises, precautions during recovery, and injury-specific rehabilitation strategies. One athlete described the value of learning from a teammate who had experienced the same injury:

*“One of my college teammates had injured the exact same area before I did, so I got a lot of advice from them on how to go through the rehab process…”*

(I001)

#### 3.1.10. Theme 10: Emotional Support

During the rehabilitation process, athletes received emotional support from parents, coaches, teammates, and rehabilitation professionals. Emotional support was primarily experienced in the form of comfort and encouragement. Many athletes described receiving comfort during periods of emotional distress. Expressions of empathy, understanding, and emotional presence from family members and teammates helped alleviate feelings of loneliness and psychological burden during rehabilitation. One participant explained:

*“It was really comforting when people around me said things like, ‘Take your time—you’ll get there, so don’t rush.’”*

(I004)

Athletes also reported receiving encouragement regarding their recovery and return to sport. Positive feedback and expressions of confidence from teammates, coaches, and rehabilitation professionals helped maintain motivation and foster hope throughout the rehabilitation process. As one athlete stated: *“My teammates told me how much they wanted me to get back to training quickly and said things like, ‘Let’s do this together.’ Their encouragement and support really gave me strength.”* (I001). Similarly, encouragement from rehabilitation professionals reinforced athletes’ confidence in their recovery progress. One participant noted: *“My rehab trainer would film my rehab sessions and show me the videos. They’d say things like, ‘You’re really getting better,’ or ‘Your body’s really coming back to life.’ Hearing those words gave me a lot of strength and motivation.”* (I002).

#### 3.1.11. Theme 11: Negative Social Influence

During the rehabilitation period, some athletes experienced negative social influences from coaches and teammates. These experiences were primarily characterized by blame and pressure to return to sport before full recovery. Some athletes reported being blamed for their injuries or having their rehabilitation efforts questioned by those around them. Such experiences intensified emotional distress and contributed to feelings of frustration and disappointment. One participant explained:

*“When I said I was going to the rehab center, some seniors and teammates would joke, ‘Going on another vacation?’ Comments like that were mentally tough to hear. I’ve been through a lot of rehab since middle school, so I know just how hard it actually is. Honestly, I think rehab exercises are even harder than regular training. So hearing people talk like that really made me feel upset.”*

(I005)

In addition, some athletes experienced pressure from coaches and teammates to return to sport before they felt physically prepared. This pressure often created additional psychological stress during the rehabilitation process. One participant noted:

*“Sometimes my coach would say things like, ‘How long are you going to keep rehabbing?’ or ‘When are you coming back?’ and ‘How are you going to compete like this?’ Those kinds of questions felt like pressure.”*

(I001)

### 3.2. Interactions Among Biological, Psychological, and Social Experiences

Participants described their biological, psychological, and social experiences as closely interconnected rather than as separate dimensions throughout the rehabilitation process (Table 5). Persistent pain and delayed physical recovery were commonly associated with anxiety regarding recovery and return to sport, whereas prolonged physical impairment was associated with uncertainty and psychological distress. In addition, psychological distress was accompanied by physical manifestations, including gastrointestinal symptoms and psychosomatic complaints such as headaches.

Participants also described interactions between social experiences and psychological experiences. Emotional support from parents, teammates, coaches, and rehabilitation professionals was associated with hope and motivation during rehabilitation, whereas positive feedback from rehabilitation professionals was associated with increased confidence in the rehabilitation process. Conversely, social pressure, including blame and expectations for an early return to sport, was associated with greater anxiety and psychological burden.

**Figure 1:**
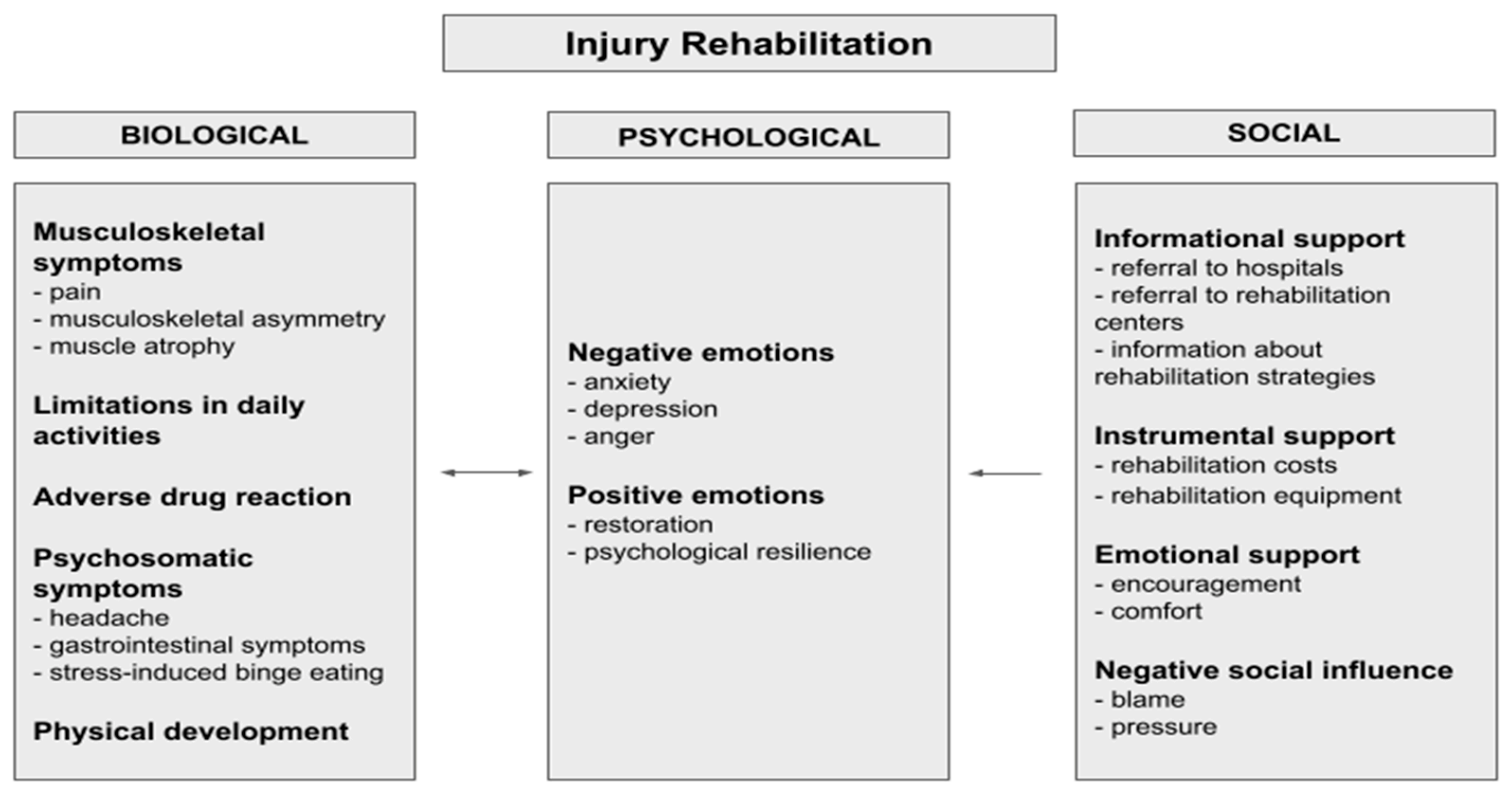
Thematic map illustrating the biological, psychological, and social experiences identified during injury rehabilitation. Legend: The figure summarizes the themes identified through inductive analysis. Bidirectional arrows indicate the interconnections among the biological, psychological, and social domains as perceived by participants and are intended to illustrate thematic relationships rather than causal pathways.

To facilitate interpretation of the qualitative findings, Figure 1 provides a thematic overview of the biological, psychological, and social experiences identified through the inductive analysis and their perceived interconnections during injury rehabilitation.

## 4. Discussion

This study qualitatively explored the biopsychosocial experiences of elite athletes and the interactions among these experiences during the injury rehabilitation process. Biological experiences were closely interconnected with psychological experiences. Persistent pain, delayed recovery, and physical impairment were commonly accompanied by negative emotional responses, including anxiety, psychological distress, and uncertainty about recovery. In turn, some athletes described psychological distress as being accompanied by psychosomatic symptoms such as headaches, indigestion, and heartburn. Stress can suppress the parasympathetic nervous system while activating the sympathetic nervous system. These physiological changes may lead to physical symptoms such as indigestion, palpitations, and elevated blood pressure (Ziegler, 2012). Therefore, it may be valuable to assess not only the physical symptoms experienced by injured athletes, such as headaches and gastrointestinal discomfort, but also the accompanying psychological factors that may contribute to these conditions.

Beyond psychosomatic symptoms, negative emotional responses have also been associated with poorer rehabilitation outcomes (Ivarsson et al., 2017; Ardern et al., 2013). Therefore, it could be valuable to assess anxiety, depression, and anger in athletes undergoing rehabilitation and to consider strategies aimed at reducing these negative emotional responses. Evidence-based psychological interventions such as cognitive behavioral therapy (CBT) and acceptance and commitment therapy (ACT) have been shown to be effective in this regard. CBT helps reduce anxiety by addressing maladaptive automatic thoughts (Zayfert & Becker, 2019), and has also been found to alleviate negative emotions in injured athletes during the rehabilitation process (Podlog et al., 2020). Acceptance and Commitment Therapy (ACT) also supports athletes in accepting negative emotions with psychological distance while promoting commitment to present tasks (Hayes & Strosahl, 2004). ACT may facilitate psychological recovery and enhance rehabilitation adherence among injured athletes (Mahoney & Hanrahan, 2011). Other psychological interventions such as positive self-talk, relaxation techniques, and goal setting can also contribute to the recovery process following sports injuries (Gennarelli et al., 2020). In the present exploratory study, none of the participants reported receiving structured psychological interventions during the rehabilitation process despite experiencing psychological distress. Considering the effectiveness of these interventions reported in previous studies, their incorporation into injury rehabilitation programs may provide additional support for the psychological recovery of injured athletes.

In addition, social experiences were closely associated with psychological experiences in the present exploratory study. Emotional support from family members and teammates was commonly accompanied by increased hope and motivation during the rehabilitation process. In particular, positive feedback from rehabilitation professionals was associated with greater rehabilitation motivation and confidence. Injured athletes often experience a range of negative emotions during rehabilitation, including anxiety, depression, and anger. Emotional support in the form of comfort and encouragement from parents, coaches, and teammates can serve as a critical psychosocial resource in overcoming these negative emotions. According to previous studies, social support contributes to emotional stability, helps alleviate anxiety related to reinjury, and ultimately enhances psychological readiness to return to sport, thereby increasing the likelihood of successful rehabilitation (Yang et al., 2014; Forsdyke et al., 2022). Taken together, these findings suggest the potential value of assessing athletes’ psychological well-being during rehabilitation and fostering emotional support from coaches, teammates, and rehabilitation professionals as part of a comprehensive rehabilitation approach.

In contrast, some athletes reported experiencing negative social influences from coaches and teammates, including blame and pressure, which were accompanied by greater psychological distress. Although injury can be one of the most distressing experiences for an athlete, instead of receiving comfort and encouragement, some athletes encountered blame or emotional neglect from those around them. Furthermore, the presence of coaches or peers who pressured them to return to play before full recovery created additional psychological stress. Such negative interactions can adversely affect athletes’ emotional well-being and, ultimately, hinder the rehabilitation process and its outcomes (Forsdyke et al., 2016). Therefore, to support the psychosocial recovery of injured athletes effectively, it may be beneficial to closely examine their emotional relationships with those around them and consider interventions that promote positive and supportive social connections. The level of social support can be quantitatively assessed using validated instruments such as the Multidimensional Scale of Perceived Social Support (MSPSS) (Zimet et al., 1990). The use of such tools allows for the identification of athletes with insufficient social support, enabling the application of tailored intervention strategies appropriate for each athlete’s context.

One noteworthy finding of the present exploratory study was that injury rehabilitation was characterized not only by negative experiences but also by positive experiences. Some participants reported physical growth and development as they progressed through their rehabilitation. Injuries are often caused by muscle weakness or muscular imbalances in specific areas (Kwan et al., 2021). Accordingly, rehabilitation programs are designed to focus on strengthening previously weakened muscles and include exercises aimed at correcting musculoskeletal asymmetries (Brukner & Khan, 2017). Participants in the present study perceived that these rehabilitation efforts contributed not only to recovery from injury but also to improvements in physical function and athletic performance. The findings of the present study are consistent with previous research suggesting that injury rehabilitation may provide opportunities for growth and positive adaptation beyond physical recovery (Galli & Vealey, 2008).

The physical development observed during rehabilitation was accompanied by positive psychological adaptations. Consistent with these physical improvements, some athletes demonstrated psychological resilience by choosing to overcome the setback of injury and aiming to return with enhanced performance. Resilience refers to the human capacity to adapt successfully in the face of adversity, trauma, tragedy, threats, or significant sources of stress (Newman, 2005). According to previous research, resilience has been shown to positively influence the recovery process of athletes who have experienced sports-related concussions (Ernst et al., 2022). Additionally, a high level of perceived legitimacy of rehabilitation, motivation, and self-efficacy has been found to facilitate adherence to rehabilitation protocols (Goddard et al., 2021). Together, these findings suggest that injury rehabilitation may serve not only as a process of recovery from injury but also as an opportunity for both physical and psychological growth. In light of these findings, structured psychosocial intervention programs or coaching strategies may help foster resilience and maintain positive psychological states in athletes during the injury rehabilitation process.

Overall, this exploratory qualitative study demonstrated that elite athletes perceived biological, psychological, and social experiences as closely interconnected throughout the rehabilitation process. Rather than occurring independently, these experiences were perceived to continuously influence one another during recovery. The present findings provide preliminary qualitative evidence supporting the relevance of a biopsychosocial perspective in understanding injury rehabilitation and may inform the development of more comprehensive athlete-centered rehabilitation approaches that consider biological, psychological, and social factors together.

All participants in the present study were elite athletes from South Korea. The Korean elite sport system has traditionally emphasized intensive athletic training from an early stage, with many young athletes prioritizing sport participation throughout their development. This sporting environment may contribute to the development of a strong athletic identity, making sports injury not only a physical setback but also a potential threat to athletes’ sense of self and athletic role. Such contextual characteristics may partly explain the psychological distress, including anxiety, frustration, and emotional difficulties, reported by participants during rehabilitation. Accordingly, the biopsychosocial experiences identified in this study should be interpreted within the sociocultural context of the Korean elite sport system. Therefore, caution is warranted when transferring these findings to athletes from different cultural or sporting environments, where developmental pathways, athletic identity, and rehabilitation contexts may differ.

This study has several limitations. First, given the exploratory qualitative nature of this study and its relatively small sample size, the findings should be interpreted within the context of the participants’ experiences rather than as being statistically or populationally generalizable. Second, all participants were recruited from a single sports university in South Korea, which may limit the transferability of the findings to athletes from different cultural, competitive, or institutional contexts. Third, the participants represented diverse sports, injury sites, rehabilitation durations, and ages. Although this heterogeneity enabled the exploration of a broad range of rehabilitation experiences, the findings should be interpreted within the context of these diverse participant characteristics. Fourth, because the interviews relied on participants’ retrospective accounts, recall bias cannot be completely excluded. Finally, although several strategies were implemented to enhance reflexivity and trustworthiness, including maintaining a non-judgmental interviewing approach and regularly discussing emerging codes with an independent researcher, the possibility of researcher influence during data collection and interpretation cannot be entirely eliminated.

Future research should include athletes from a wider range of sports, competitive levels, and cultural settings to further examine the transferability of the findings. Longitudinal qualitative studies following athletes across different stages of rehabilitation would also provide a more comprehensive understanding of how biological, psychological, and social experiences evolve over time. In addition, incorporating multiple perspectives, such as those of coaches, physiotherapists, physicians, and family members, would enable methodological triangulation and provide a more comprehensive understanding of athletes’ injury rehabilitation experiences.

## 5. Conclusions

This exploratory study examined elite athletes’ rehabilitation experiences from biological, psychological, and social perspectives and explored the interactions among these domains. Physical symptoms, such as pain, muscle atrophy, and delayed recovery, were associated with negative emotional responses, including anxiety. Conversely, negative psychological responses appeared to contribute to psychosomatic symptoms, such as headaches and indigestion. Emotional support, including comfort and encouragement, was associated with positive emotional experiences, whereas negative social influences, such as blame and pressure, were associated with negative emotional responses. In addition to negative experiences, athletes also described positive psychological experiences during rehabilitation, including the development of resilience. These findings suggest that injury rehabilitation is a dynamic biopsychosocial process in which biological, psychological, and social factors interact throughout recovery.

Based on these findings, an integrated support system that addresses not only physical treatment but also psychological well-being and social support may be beneficial during athletes’ rehabilitation. Implementing psychological interventions to reduce negative psychological responses may be beneficial for athletes during the rehabilitation process. Furthermore, strengthening emotional support and developing educational and psychosocial intervention programs to reduce negative emotions and adverse social influences may contribute to more comprehensive rehabilitation.

## Figures and Tables

**Table 1:** Inclusion and exclusion criteria.

Inclusion	Exclusion
1. Elite athletes who have experienced an injury 2. Athletes who were unable to train or compete for more than three months due to injury 3. Athletes who have undergone rehabilitation for more than three months following injury	1. Athletes who declined to participate in the study

**Table 2:** Participants in the in-depth interviews.

	Sex/Age	Sport/Years of Experience	Injury Site	Duration of Rehabilitation
I001	F/32	Judo/12	Knee	12 month
I002	M/31	Fencing/20	Hip joint	9 month
I003	M/31	Ski/20	knee	12 month
I004	F/26	Athletics/9	Knee	6 month
I005	F/26	Field hockey/15	Spine	3 month

**Table 3:** Example of the inductive categorization process.

ID	Raw Data	Code	Theme	Category
I001	When I was stressed, I tended to eat spicy or stimulating foods, and the next day I would experience stomach discomfort and pain. Then, when I felt stressed again, I would return to eating those foods. I think I kept repeating this pattern	stress-induced binge eating	psychosomatic symptoms	biological perspective
I001	Because I was constantly worried, I found myself frowning and feeling tense without realizing it. As a result, I experienced frequent headaches.	headache	psychosomatic symptoms	biological perspective
I001	My teammates told me they wanted me to return to training as soon as possible and encouraged me by saying we would do it together. Their support and encouragement gave me strength.	comfort	emotional support	social perspective
…	…	…	…	…

**Table 4:** Major Themes from a Biopsychosocial Perspective on Elite Athletes’ Experiences During Injury Rehabilitation.

Code	Theme	Category
pain musculoskeletal asymmetry muscle atrophy	musculoskeletal symptoms	biological perspective
limitations in daily activities	limitations in daily activities	
gastrointestinal symptoms	adverse drug reaction	
headache gastrointestinal symptoms stress-induced binge eating	psychosomatic symptoms	
physical development	physical development	
anger anxiety depression	negative emotions	psychological perspective
restoration psychological resilience	positive emotions	
referral to hospitals referral to rehabilitation centers information about rehabilitation strategies	informational support	social perspective
rehabilitation costs rehabilitation equipment	instrumental support	
encouragement comfort	emotional support	
blame pressure	negative social influence	

**Table 5:** Interactions among Biological, Psychological, and Social Experiences.

Interaction Pathway	Representative Quotation	Description of Interaction
Biological to Psychological	*“There was always a warm sensation around the surgical site. I kept thinking, ‘What if it’s getting inflamed again?’ Those thoughts made me feel anxious because I worried that my recovery was slower than expected.”* (I001)	Persistent pain and delayed physical recovery were associated with anxiety regarding recovery and return to sport.
Biological to Psychological	*“Since the injury just wasn’t getting better, I started thinking, ‘What if I can’t do fencing anymore?’ Those thoughts made me feel really uneasy.”* (I002)	Prolonged physical impairment was associated with uncertainty and psychological distress.
Psychological to Biological	*“When I was stressed, I’d eat spicy or greasy food. The next day my stomach would hurt, but when I became stressed again, I repeated the same behavior.”* (I001)	Psychological stress was accompanied by gastrointestinal symptoms.
Psychological to Biological	*“Since I was always under stress, I often experienced indigestion and migraines during rehabilitation.”* (I004)	Emotional distress was accompanied by psychosomatic symptoms.
Social to Psychological	*“Their encouragement and support really gave me strength during rehabilitation.”* (I001)	Emotional support was associated with increased hope and motivation during rehabilitation.
Social to Psychological	*“Hearing those words from the rehabilitation therapist gave me a lot of strength and motivation to continue.”* (I002)	Positive feedback from rehabilitation professionals was associated with increased confidence and rehabilitation motivation.
Social to Psychological	*“People kept asking me, ‘How long are you going to keep rehabbing?’ Those kinds of questions felt like pressure.”* (I001)	Social pressure was associated with increased anxiety and psychological burden.

## Data Availability

The datasets generated and/or analysed during the current study are not publicly available because of the decision and recommendations of the Institutional Review Board of H+ Yangji Hospital. However, the data are available from the corresponding author upon reasonable request.

## Abbreviations

The following abbreviations are used in this manuscript:

ACT	Acceptance and Commitment Therapy
ACL	Anterior Cruciate Ligament
CBT	Cognitive Behavioral Therapy
